# Refractive changes in patients with autoimmune scleritis

**DOI:** 10.1007/s12348-011-0023-0

**Published:** 2011-04-05

**Authors:** Lourdes Arellanes-Garcia, Maria del Carmen Preciado-Delgadillo, Manuel Garza-Leon

**Affiliations:** 1 Clinic of Inflammatory Eye Disease, “Dr. Luis Sanchez Bulnes” Hospital Asociación para Evitar la Ceguera en México IAP, Mexico, Mexico; 2 Instituto para Preservación de la Visión, Monterrey, Nuevo Leon Mexico

## Introduction

Scleritis is a severe inflammatory disease characterized by edema and inflammatory cell infiltration of the sclera [1, 2]. Without treatment, the condition may be progressively destructive, leading sometimes to loss of vision or loss of the eye [1, 2].

About 30–50% of all scleritis patients have an associated systemic disease [1]. Most common associations are: rheumatoid arthritis (50–60%) and primary systemic vasculitis like Wegener’s granulomatosis (20%) followed by inflammatory bowel disease (15%) [1].

Complications have been reported in almost 60% of scleritis patients [1, 2]. In their series, Jabs et al. found decreased visual acuity in 15.9% of patients during the inactive phase of the disease [1]. Sainz de la Maza et al. described this complication in 37% of their patients [2].

Some authors have reported transient myopia during the acute phase of anterior diffuse [3] and necrotizing scleritis [4]. In one report transient myopia developed after cataract surgery in a patient with necrotizing scleritis [5]. Secondary astigmatism in patients with necrotizing scleritis has also been found. The authors associated this finding to scleral thinning [6].

To the best of our knowledge, no attempt to systematically characterize the refractive changes associated to scleritis has been done. Our purpose was to determine the clinical characteristic, scleral rigidity, and refractive changes in a cohort of scleritis patients who were followed up since admittance until resolution of inflammation.

## Material and methods

This protocol was reviewed and approval by the hospital ethics committee and has been performed in accordance with the ethical standards laid down in the 1964 Declaration of Helsinki. All the patients gave their informed consent prior to their inclusion in the study.

We included patients of the Inflammatory Eye Diseases Clinic of the “Dr. Luis Sánchez Bulnes” Hospital, with a diagnosis of noninfectious active anterior scleritis with or without associated systemic disease. Immunosuppressive treatment was prescribed according to the associated disease. All patients signed an informed consent form to be included in the study. Demographic data of patients were obtained. Type and location of scleral inflammation and associated systemic disease were also recorded. Best corrected visual acuity (BCVA), spherical and cylindrical error, intraocular pressure (IOP), and scleral rigidity were measured. Initially, an auto-refraction was made (Topcon RM-A2300) followed by a mono-ocular subjective refraction with Snellen chart, spherical power was determining by the red–green test, and the cylinder by the cross cylinder technique. Because all the patients were adult, cycloplegic refraction was not realized. The scleral rigidity coefficient was determinate according to Friedenwald’s nomogram using a Schiotz tonometer [7]. Briefly, after 15 min on supine decubitus and previous application of topical anesthetic, IOP was measured with the Shiotz tonometer, with 5.5 and 7.5 weights; there is a modification of the technique, where these two measures are compared with the IOP taken with Goldman tonometer; finally, in the Friedenwald’s nomogram, we searched the point where both IOP measurements intersect. All measurements were done by the same ophthalmologist (MCPD) [7, 8].

Patients were evaluated during the active and inactive phases of disease. Minimum follow-up time was 6 months.

Central tendency statistical of data were obtained. Wilcoxon ranked signed test was performed to compare initial and final data of BCVA, IOP, refractive error, and coefficient of scleral rigidity. Statistically significant *p* value was <0.05.

## Results

Four female patients (seven eyes) were included. Average age at admission was 54 years (±3 SD). Mean follow-up time was 20 months (±4 SD). Wegener’s granulomatosis was diagnosed in two patients, rheumatoid arthritis in one, and the fourth had a positive serology for the HLA-B27 antigen. Diffuse scleritis was found in one eye and nodular scleritis in six eyes. Most common nodule location was superior (three eyes): Inferior, nasal, and temporal locations were found in one eye each.

When we compared values during the active and inactive inflammation (Table 1), we found that in four eyes the cylindrical power increased at least 1D, and that the axis changed at least 10°. In one eye there was a myopic shift of 1D. All patients had a scleral rigidity coefficient lower than the value reported as normal by Friedenwald (0.0215). This reduction was found in both the active (0.0197) and inactive phases (0.0118). A statistically significant difference in cylindrical axis and scleral rigidity coefficient was found when we compared them during active and inactive inflammation stages. These results must be taken with precaution due to the small number of patients.

**Table 1:** Clinical, refractive and scleral rigidity characteristics

Variable	Active phase, mean (range)	Inactive phase, mean (range)	*P* value
Visual acuity	20/30 (20/50–20/20)	20/30 (20/50–20/20)	0.83
Intraocular pressure (mmHg)	11.71 (10–16)	13.0 (12–15)	0.60
Sphere (D)	−0.50 (+4.00 to −4.75)	+1.75 (+5.00 to −3.75)	0.17
Cylinder (D)	−4.07 (−0.75 to −6.00)	−4.10 (−0.75 to −6.00)	0.08
Axis cylinder (degrees)	110.8 (9–174)	59 (4–170)	0.02
Scleral rigidity^a^	0.0197 (0.0177–0.0244)	0.0118 (0.0053–0.0183)	0.02

^a^Average scleral rigidity coefficient according to Friedenwald’s nomogram

## Discussion

We are unaware of previous reports evaluating refractive changes in patients with scleritis. Jabs et al. reported an associated systemic disease in 27% of their group of patients. The authors did not find any statistically significant association between final visual acuity and the presence of corneal alterations, cataracts, posterior segment complications, or intraocular inflammation. The refractive status of those patients was not evaluated [1].

Some considerations must be taken into account when analyzing the results, the most important is the small number of patient studied, also the lack of corneal topographic changes measured.

Comparing the initial (active inflammation) and final (inactive inflammation) values in our group of patients, we found in four eyes an increase of at least 1D in the cylindrical power, in these cases, the cylindrical axis changed at least 10°. In one eye a 1D myopic shift was found.

In all the studied eyes, there was a 10–15% decrease in the scleral rigidity coefficient in the final measurement, in all cases this value was lower than the normal value reported by Friedenwald [7].

A statistically significant difference in cylindrical axis and scleral rigidity coefficient was found during the active and inactive inflammation phases. Our findings suggest that the modification of the scleral rigidity induces an alteration in the stability of the corneal contour at the limbus. This scleral–corneal interaction was reported by Newton and Meek in 1998 [9]. They described that at the limbus, the deep fibers of the sclera tighten to form the scleral spur, a rigid ring structure that together with the corneal annulus (which is formed by a circumferential swath of limbal fibrils originating in the cornea) probably accounts for the stability of the corneal contour.

Inflammation of the sclera seems to alter the scleral structure. Fibroblastic transformation, breakdown of proteoglycan linkages, and degeneration of collagen fibrils have been found in the inflamed sclera [10], these alterations may permanently modify the scleral structure and rigidity. If this change affects the scleral spur and consequently the corneal annulus, an alteration of the stability of the corneal contour may be expected, explaining the modification in the cylinder axis, observed in our cases.

We have hypothesized that the initial (active phase) increased thickness and scleral edema (5) producing a force in the limbus produce changes in the cornea, shape, and in the inactive phase, the decrease of the strength of the limb associated with the loss of scleral tissue (5) and the secondary loss of structural support in the necrotic area of the limbus could work like a belt that exerts its force centripetally, allowing the central dome of the cornea to be displaced anteriorly, thus increasing its curvature.

## Conclusions

In this small group of patients with autoimmune anterior scleritis, we observed a modification of the scleral rigidity coefficient values and refractive changes during the course of the disease. These alterations may explain some cases of decreased VA, in patients with inactive scleral inflammation that is not explained by corneal abnormalities, cataracts, posterior segment complications, or intraocular inflammation.
